# Migration and social vulnerability: impact on the life and health of the refugee population

**DOI:** 10.1590/1980-220X-REEUSP-2023-E002en

**Published:** 2024-03-18

**Authors:** José Manuel Peixoto Caldas

**Affiliations:** 1 Universidade Federal da Paraíba, Centro de Ciências Aplicadas e Educação, Departamento de Ciências Sociais, Rio Tinto, PB, Brazil.; 2 Universidad de Salamanca, Instituto de Iberoamérica, Salamanca, España.

The present special issue, consisting of original articles, systematic and integrative reviews, reflections and experience and case reports, seeks to give visibility to studies and research that delve into the intersection of migrants and refugees with: gender, generation, ethnicity, violence, public policies, among others. In addition, it aims to disseminate work that analyzes and evaluates health services, in the forms of reception and monitoring of populations in situations of social vulnerability in which refugees find themselves.

We all know that migratory flows are usually identified as a public health challenge, making it important (and necessary) to understand the impacts of migration on health, both from the perspective of the health systems in the countries that receive immigrants, and from the perspective of immigrant and non-immigrant populations living in these contexts.

In this field, the World Health Organization^(1)^ emphasizes four principles that public health should promote in order to achieve the health of immigrants and the population of host societies: (1) disparities between immigrants and non-immigrants in terms of health status and access to health care should be avoided; (2) the right to health protection for migrants should be guaranteed, reducing discrimination and barriers that may exist to immigrants’ access to health; (3) the mortality and morbidity of migrant populations should be reduced; and (4) the negative impacts of the migratory process should be minimized, particularly those that lead to greater vulnerability and health risks for migrants, regardless of the host context.

However, despite the growing recognition of the importance of this issue, there are still gaps in our knowledge of the relationship between migration and health, and of the real impacts of migration on health.

The study of the relationship between immigration and health has been seen from two fundamental perspectives: on the one hand, the health status of migrants compared to nationals of the host countries and, on the other hand, their access to health care in the destination countries:

(1) Studies analyzing health status indicators, comparing immigrants with natives of host countries, identify that immigrants tend, in the initial phase (as newcomers to destination countries), to report better health status than natives or populations of the same origin who have lived in host countries for longer; in a later phase, after years of residence in the host society, they report or perceive lower levels of health than the native population^(2)^. The majority of available studies suggest that some immigrant groups tend to be more vulnerable to diseases and health problems. The conditions in which migration takes place and the health determinants associated with the migratory process or integration into the host society often reflect social inequalities that contribute to greater vulnerability to disease: poorer socio-economic and working conditions, worse housing conditions, which is reflected in extremely vulnerable lifestyles, difficulties in contacting administrative and legal systems^(3)^. At the same time, the stigmatization of immigrants or discrimination based on their ethnic or racial origin often has an impact on the state of health and well-being of immigrant populations in host contexts^(4)^.
(2) The access and use of health care by immigrant populations are recognized as important indicators of integration into host societies, and are also fundamental to the morbidity of these populations, their health and well-being. In this context, managing the health and promoting the well-being of immigrant populations has meant that health systems must ensure accessibility and respond adequately to their needs. However, several studies have concluded that immigrant populations are often not covered or adequately covered by the health systems of host countries^(2)^.

In this context, research in the area of health and immigration has sought to understand the conditions of access to and use of health services, identifying the factors that promote or inhibit their use, considering the influence of both individual factors (sociodemographic characteristics, attitudes and beliefs towards health and illness, knowledge of health rights, language difficulties) and contextual or structural factors in the host society (legal and institutional contexts of access to and provision of health care for immigrants, the role of professionals and the characteristics of health services)^(2,5,6)^.

As a result, there are still many structural socio-political, administrative, ethnic and cultural obstacles to achieving true global citizenship, which is why many of the authors in this issue argue that health policies explicitly geared towards immigrants are necessary in order to reduce inequalities, but also by making health systems more immigrant-friendly in other ways, such as overcoming cultural and linguistic gaps, improving the intercultural competences of health professionals and organizations, increasing the health literacy of immigrants^(6,7)^, and taking ethnic-cultural diversity into account.

## References

[1] Entidade Reguladora de Saúde. (2015). Acesso a cuidados de saúde por imigrantes..

[2] Ingleby D. (2016). Access to health service for migrants: what are the policy challenges? Lessons from the MIPEX study: David Ingleby.. Eur J Public Health..

[3] Caldas JMP, Oliveira MB, Manso AG, Topa JB, Vicente IS, Vicente MC, Centeno Martín H (2023). Procesos migratorios y desafíos en el marco del pacto mundial para la migración segura, ordenada y regular..

[4] Oliveira CR, Gomes N (2018). Migrações e Saúde em números: o caso português..

[5] Almeida LM, Costa-Santos C, Caldas JP, Dias S, Ayres-de-Campos D (2016). The impact of migration on women’s mental health in the postpartum period.. Rev Saude Publica..

[6] Ortiz Scaglione MA (2016). A saúde das mulheres imigrantes: Uma questão de cidadania e inclusão [tese]..

[7] Barrientos DMS, Méric OGA, Romero LL, Santos NT, Neia CM, Lins ALDFP (2023). Captar y comprender la realidad de los inmigrantes sudamericanos a partir del análisis del discurso.. Contribuciones a Las Ciencias Sociales..

